# Reconstruction Accuracy vs. Discriminative Power: Spectral Unmixing Performance in Brain Tissue Hyperspectral Imaging

**DOI:** 10.3390/bioengineering13070835

**Published:** 2026-07-21

**Authors:** Alejandro Martinez de Ternero, Alberto Martín-Pérez, Manuel Villa, Eduardo Juarez

**Affiliations:** CEIMM, Center for Industrial Electronics and Multimodal Systems, Universidad Politécnica de Madrid, 28031 Madrid, Spain; a.martinp@upm.es (A.M.-P.); manuel.villa.romero@upm.es (M.V.); eduardo.juarez@upm.es (E.J.)

**Keywords:** spectral unmix, classification, brain tissue, hyperspectral

## Abstract

Hyperspectral imaging holds promise for intraoperative brain tissue characterisation, but its high dimensionality complicates clinical deployment. Spectral unmixing offers a pathway to compress data into interpretable abundance maps, yet its impact on downstream tissue discrimination remains unclear. This study evaluated four unmixing models (ASM, LMM, PPNMM, FBM) and three normalisation strategies (SNV, $L_{2}$, Max) across two hyperspectral in vivo brain surgery datasets HELICoiD (*n* = 15), SLIMBRAIN (*n* = 27). Spectral reconstruction (SAM) and tissue classification (F1-score) were assessed using optimised SVMs trained on abundance vectors versus full spectra. SNV normalisation consistently yielded superior classification performance, while the Absorption and Scattering Model (ASM) achieved median PSNR improvements exceeding 15 dB over linear models due to its scattering correction term. Notably, ASM-SNV abundance maps maintained classification accuracy comparable to full-spectrum classifiers despite an order of magnitude data compression. Conversely, superior reconstruction fidelity did not guarantee improved discrimination: ASM-Max significantly degraded tumour classification in SLIMBRAIN (p<0.001), indicating that task-agnostic reconstruction can discard clinically relevant spectral features. Tumour classification remained the most challenging task across all conditions, reflecting inherent inter-tumour spectral heterogeneity. These findings establish ASM-SNV as the best tested configuration for intraoperative guidance, balancing spectral interpretability and classification robustness while highlighting the need for task-optimised unmixing frameworks to address pathological variability.

## 1. Introduction

Hyperspectral Imaging (HSI) captures detailed spectral information about tissue composition that is often invisible to the naked eye [1,2,3]. In neurological applications, this has the potential to improve the detection and monitoring of conditions ranging from brain tumours to vascular diseases [4,5,6]. These distinctions arise from measurable shifts in tissue composition and microstructure. Pathological tissues typically exhibit altered cellular organisation, changes in the concentration and redox state of primary chromophores (oxyhemoglobin, deoxyhemoglobin, and cytochrome c oxidase), and variations in water and lipid content. Tumours further demonstrate altered vascularisation and increased metabolic demand, collectively modifying absorption peaks and scattering coefficients. However, the high dimensionality of HSI data presents significant challenges; each spatial pixel contains tens to hundreds of spectral bands. This increases computational costs and creates redundant information [7], which complicates data analysis and risks overfitting in machine learning algorithms, particularly when dealing with limited clinical datasets [5].

Existing dimensionality reduction techniques address this redundancy through projection and optimisation methods [8]. Classical methods such as Principal Component Analysis (PCA) and Independent Component Analysis (ICA) reduce dimensionality by projecting spectra onto subspaces that capture variance or statistical independence [9]. Although computationally efficient, these methods suffer from an interpretability gap because the components are arbitrary linear combinations of all input bands rather than physical tissue properties [10]. Similarly, manifold learning methods [11,12,13] and optimisation techniques produce embeddings that are difficult to map back to the original spectral space. Deep learning models, such as CNNs [14,15] offer better feature extraction but remain black boxes that require extensive data to train. In conclusion, these techniques often suffer from a lack of interpretability, where compressed information can only be used to reconstruct the signal, or a dependence on the choice of the learning algorithm, which limits their generalisation to different datasets [16].

A growing trend of research explores the potential of Spectral Unmixing (SU) to extract meaningful information from HSI data by decomposing the observed spectrum into a combination of known spectra from existing materials, called endmembers [17,18,19]. By applying this method, HSI information can be effectively reduced to a set of statistical values called abundance maps, which relate to the concentration of each material in the capture. These abundance maps not only compress the high-dimensional data into a compact, physically interpretable representation but also contain important information for tissue classification and intra-operative guidance, since choosing a set of representative endmembers allows the visualisation of information such as total haemoglobin (HbT) or oxygen saturation (StO2) maps [20] in the form of pseudo-colour images. Although HSI captures rich biochemical information, its high dimensionality and sensitivity to illumination variations hinder clinical deployment, motivating SU as a strategy to compress data into physiologically interpretable abundance maps. Prior work has primarily focused on maximising reconstruction fidelity or accelerating computation, leaving a critical gap in understanding whether high reconstruction accuracy actually preserves the discriminative spectral features required for downstream clinical tasks [5,21,22].

To address this gap, this study evaluates two publicly available databases: HELICoiD [23] and SLIMBRAIN [24] using spectral unmixing techniques under several common HSI normalisation schemes. For each normalisation and SU model, reconstruction errors are analysed at a per-band and per-tissue-type level to identify cross-dataset trends. Finally, the discriminative utility of the resulting abundance maps is assessed by training a supervised classifier on the abundance features and evaluating its classification accuracy. This workflow demonstrates whether the compressed abundance space preserves sufficient tissue-specific information for reliable downstream analysis.

## 2. Materials and Methods

The following subsections present the two HSI datasets employed in this work, including details on the acquisition hardware, the pre-processing pipeline applied to the raw measurements, and the annotation protocol used to generate ground truth tissue labels. In addition, they describe the various SU methods evaluated in this study, together with the unmixing framework within which they were applied.

### 2.1. Data Description

On the one hand, the HELICoiD database comprises a set of in vivo HSI images at different stages of a brain tumour resection operation for 22 different patients. The prototype created to capture the HSI information used a pushbroom camera that captures in the visual and near-infrared area (VNIR), from 400 to 1000 nm, coupled with a line-focused light source that provided enough power in the same spectral range. The camera was able to capture spectral information with a resolution of 2–3 nm, resulting in a total of 826 spectral bands. More details on this database can be found in [23].

On the other hand, the SLIMBRAIN database is a multimodal brain tissue database created also during in vivo brain tumour resection operations for more than 193 patients. The prototype comprises two hyperspectral cameras, one snapshot camera that works in the near infrared (NIR) from 650 to 950 nm with 25 wavelengths, and a linescan camera capable of capturing the 400–1000 nm range with a total of 369 spectral bands. A depth camera is also present in the prototype, so that the 3D surface is also recovered from the scene. In this case, two fibre optic cables homogeneously illuminate the craniotomy site, serving as the light source for both the snapshot and linescan cameras. The camera chosen for this study is the linescan model, as it delivers data within the same range as the HELICoiD system and relies on comparable technology.

Acquisition duration is constrained by the mechanical scanning process and the exposure time required to obtain an adequate signal-to-noise ratio. The HELICoiD pushbroom system requires at least one minute to acquire a hyperspectral cube, while the SLIMBRAIN line-scan protocol requires approximately 80 s. Because these acquisitions are performed during relatively static phases of surgery (e.g., after dural opening or during resection planning), they can generally be incorporated into the operative workflow without requiring continuous interruption of the surgical procedure.

Captures exhibiting motion artefacts, excessive specular reflection, or poor pathological exposure were excluded, resulting in a final cohort focused on regions where the healthy cortex, tumour, or blood vessels were clearly visible. As shown in Table 1, both datasets are inherently imbalanced, with healthy tissue and vascular structures dominating the pixel distribution while tumour classes remain under-represented. Notably, SLIMBRAIN contains approximately one order of magnitude more labelled pixels than HELICoiD providing a larger sample for the cross-dataset validation. The complete list of capture IDs selected from each database is listed in Appendix A.

### 2.2. Capture and Annotation Protocol

The acquisition procedure for both databases is comparable: neurosurgeons remove a portion of the skull and carefully open the thin tissue layer known as the dura mater to expose the cortical surface of the brain. Subsequently, an HSI scan is performed, during which the pathological region is visible on the exposed brain surface. In addition, some captures are also produced after tumour resection.

In both databases, ground truth labels were generated by the operating neurosurgeon using a custom segmentation tool [23] after the operation. The tool allows the neurosurgeon to delineate contiguous tissue regions and assign different classes based on a spectral metric, ensuring that spectral consistency is achieved for the same tissue classes. For this study, tissue classes were grouped into healthy cortex, pathological tumour, and blood vessels, excluding non-biological materials such as surgical instruments or drapes. In addition, in some captures, neurosurgeons placed sterile rubber rings that indicate areas of pathological or healthy tissue to help in the labelling process. It is important to note that these labels reflect intraoperative clinical assessment rather than histopathological verification. Intraoperative biopsies of healthy tissue or tumour margins are rarely obtained due to invasiveness and surgical risk, which introduces inherent subjectivity into the annotation process. While this aligns with current clinical workflow, it highlights a need for improved ground truth validation, particularly at tumour margins. Emerging initiatives, such as the STRATUM-3D project [25], are addressing this by synchronising intraoperative labelling with targeted biopsies and ex vivo hyperspectral imaging of resected tissue validated by pathologists to establish more precise spectrum-pathology correlations.

**Figure 1:**
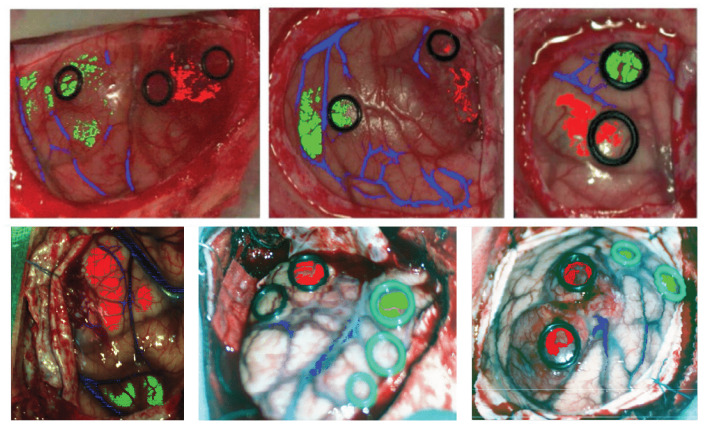
Synthetic RGB images with overlapped labels from HELICoiD (**top**) and SLIMBRAIN (**bottom**) datasets. Green, red and blue indicate healthy tissue, pathological tissue and blood vessels, respectively, as annotated by the neurosurgeon. Sterile rubber rings are placed in some captures to help the post-labelling process.

Figure 1 presents three pseudo-RGB images of representative patients in the HELICoiD (top row) and SLIMBRAIN (bottom row) datasets. The ground truth annotations provided by the neurosurgeons are overlaid on each image. Healthy tissue is represented in green, the tumour in red, and the blood vessels in blue.

### 2.3. Hyperspectral Pre-Processing

**Figure 2:**
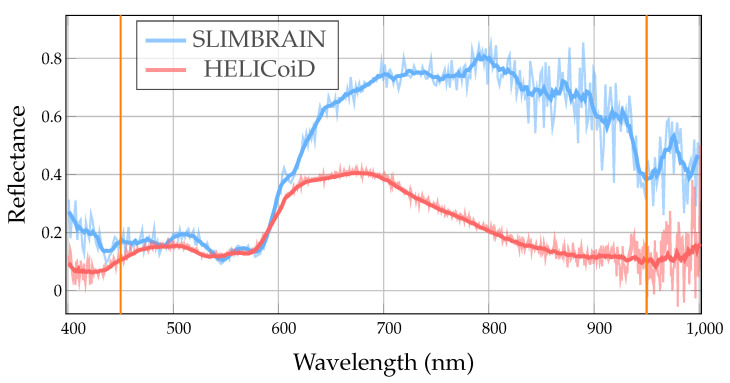
Reflectance values collected from a healthy pixel of a representative patient for each dataset, where the transparency line indicates the original signal and the solid line is obtained after the denoising process step. The vertical orange lines define the crop to remove the first and last bands that contain too much noise.

A standardised pre-processing pipeline was applied to all datasets to suppress non-biological variance while preserving spectral shape, which is critical for both reconstruction accuracy and downstream classification. Raw hyperspectral measurements as radiance values L(λ) at each wavelength λ and spatial pixel. Since illumination, sensor gain, and optical path may differ between experiments, it is crucial to normalise the radiance so that the resulting measurements are comparable across all acquisitions. This is conducted by transforming the radiance values into reflectance R(λ) using a white ceramic reference captured under the same conditions, as described in Equation (1)

(1) $$R\left(\lambda \right)=\frac{L\left(\lambda \right)-L_{dark}\left(\lambda \right)}{L_{white}\left(\lambda \right)-L_{dark}\left(\lambda \right)}$$

where $L_{white}\left(\lambda \right)$ is the measured radiance of the white calibration plate and $L_{dark}\left(\lambda \right)$ is the radiance measured with the sensor aperture closed, which accounts for sensor dark current and electronic read noise. Following reflectance conversion, a spectral crop was applied between 450 and 950 nm to exclude regions with insufficient illumination and elevated sensor noise at the spectral edges. A uniform filter (5×5×5 kernel) was applied to mitigate linescan discontinuities and spectral highlights, since polarisation filters were absent in the cameras, followed by a Savitzky–Golay spectral filter (window size 9) to reduce high-frequency noise. Raw and cleaned spectra are visible in Figure 2, where it can also be seen that the SLIMBRAIN dataset exhibits a lower Signal-To-Noise (SNR) ratio that could be due to the choice of the illumination process, not focusing the light on the capture line of the camera. 

Subsequently, the reflectance (*R*) spectra are converted to absorbance (*A*) values to obtain a representation that is more directly related to the concentrations of biological chromophores within the tissue. This conversion employs the logarithmic expression shown in Equation (2), which is derived from the Beer–Lambert law describing light attenuation in biological media.

(2) A(λ)=−log(R(λ))

Finally, a manual spatial mask was applied to isolate biological tissue regions, preventing non-tissue elements (e.g., surgical tools, drapes) from biasing the reconstruction metrics.

### 2.4. Spectral Unmixing

Spectral unmixing methods represent observed pixel spectra as weighted combinations of physically interpretable endmembers. In this section, a range of mixing models is examined, spanning from the standard linear formulation to more complex bilinear, polynomial, and physically inspired scattering approaches. The estimation procedures for the corresponding abundance coefficients and auxiliary parameters are subsequently detailed.

#### 2.4.1. Endmember Library

Spectral unmixing requires endmembers that represent the dominant chromophores and tissue constituents in the field of view. We selected a fixed library of ten endmembers derived from established biophysical literature [26,27,28,29], using spectral characteristics from the Biomedical Optics Research Laboratory (BORL) repository [30]. We chose a fixed library over data-driven extraction methods to ensure reproducibility and physiological interpretability, since adaptive methods often yield camera- or dataset-specific signatures that degrade across institutions. A canonical library avoids this variability, enabling direct comparison of abundance maps to known tissue components regardless of acquisition context.

The library comprises ten spectral signatures grouped into three functional categories:

- Haemoglobin species: $HbO_{2}$ and Hb, the dominant visible/NIR absorbers used to quantify tissue oxygenation.
- Cytochrome redox pairs: Cytochrome *b*, cytochrome *c* oxidase, and cytochrome *aa*_3_ (oxidised and reduced forms), which track mitochondrial redox state and cellular respiration.
- Tissue constituents: Water and lipids, which account for bulk tissue composition and provide baseline NIR absorption features.

This configuration prioritises interpretability, though it inherently assumes that pathological spectral variation can be approximated by combining healthy chromophore profiles.

#### 2.4.2. Unmixing Models

**Linear Mixing Model (LMM)**

The Linear Mixing Model (LMM) is the most straightforward way to describe how a pixel spectrum (y) is generated from a set of endmembers. In the LMM, each pixel is assumed to be a linear combination of all the endmember spectra, plus additive noise [31]. Let *K* be the number of endmembers, E the endmember matrix of shape B×K that contains the spectra of the different endmembers, and $a={\left[a_{1},\ldots ,a_{K}\right]}^{T}$ the abundance vector. Then the model can be written as Equation (3)

(3) $$y=\sum_{k=1}^{K}a_{k}e_{k}+n=\mathrm{Ea}+n$$

where n represents the additive noise vector.

**Fan Bilinear Model (FBM)**

The Fan Bilinear Model (FBM) [32] extends the simple linear formulation by explicitly accounting for second-order interactions between endmembers. When multiple materials coexist within a single pixel, photons can scatter off one constituent and then off another, producing spectral signatures that deviate from a purely linear combination [33]. The FBM attempts to capture these effects by adding a bilinear term that couples every pair of endmembers. The FBM is defined in Equation (4)

(4) $$y=\sum_{k=1}^{K}a_{k}e_{k}+\sum_{j=1}^{K}\sum_{{l=1,}_{l\neq j}}^{K}a_{j}e_{j}⊙a_{l}e_{l}+n$$

where ⊙ refers to the Hadamard product (element-wise).

**Polynomial Post-Non-Linear Mixing Model (PPNMM)**

The PPNMM models non-linearity as a polynomial transformation of the linear mixture [34]. This can be achieved by splitting the abundance vector into the linear part a and a non-linearity coefficient per-pixel *b*, as reflected in Equation (5)

(5) y=Ea+b(Ea⊙Ea)+n

where b∈[−0.5,0.5] is a per-pixel coefficient controlling the strength of the quadratic term, reducing to LMM when b=0. Unlike FBM, which models pairwise endmember interactions, PPNMM applies a global non-linear transformation to the entire linear mixture.

**Absorption and Scattering Model (ASM)**

In contrast to the purely mathematical variants of LMM presented, we propose a physically motivated model that includes a correction term to account for the scattering of biological tissues, similar to that proposed in [35]. The experimental scattering properties of tissues in the visible and near-infrared range have previously been fitted as Mie scattering [26] following Equation (6)

(6) $$\mu_{s}^{\prime }=\alpha {\left(\frac{\lambda }{500}\right)}^{-\beta }$$

where α and β are the non-negative scattering amplitude and power coefficients to fit and the wavelength λ is scaled to a reference, in this case 500 nm, to be dimensionless as in [26].

In the ASM, the measured spectrum is represented as the sum of two terms: an ideal linear mixture using the LMM and a weighted wavelength-dependent scattering component as depicted in Equation (7)

(7) $$y=\mathrm{Ea}+\tau \cdot \mu_{s}^{\prime }\left(\lambda \right)+n$$

where τ is a positive attenuation factor per pixel that controls the overall contribution of scattering.

#### 2.4.3. Unmixing Framework

Spectral unmixing decomposes each measured pixel spectrum y into a combination of constituent materials, represented by the abundance vector a, using a fixed endmember library E. Depending on the mixing model, the framework optimises additional parameters to capture non-linear or physical effects, such as the bilinear coefficient *b* in the PPNMM or the scattering coefficients θ={τ,α,β} in the ASM.

The unmixing problem is formulated as a constrained optimisation task that minimises the root-mean-squared error (RMSE) between the observed spectrum and its reconstruction by the mixing model M:

(8) $$\min_{a,\theta }\;\mathrm{RMSE}\left(y,M\left(E,a,\theta \right)\right)\;\mathrm{subject}\;\mathrm{to}\;a,\theta \geq 0$$

where non-negativity constraints are applied to both a and θ to prevent physically implausible negative values.

We implemented the optimisation in PyTorch (2.6.0) to accelerate matrix operations on GPU hardware. The RMSE was minimised using the Adam optimiser (learning rate 0.25) for up to 5000 epochs, with convergence typically achieved after 2500 epochs. On an NVIDIA A100 GPU, mean processing time per hyperspectral cube ranged from 15 s for the standard LMM to 40 s for the more complex ASM, reflecting the additional computational overhead introduced by the extra parameters. Although these times demonstrate the feasibility of GPU-accelerated unmixing, they remain insufficient for real-time intraoperative guidance. Further algorithmic optimisation or deployment on dedicated embedded hardware will be required to reduce latency to clinically acceptable levels.

## 3. Results

The results section of this paper is organised into two main parts. In the first part, we assess the impact of normalisation and the selection of spectral unmixing models by analysing how accurately the reconstructed spectra, obtained from the estimated abundances, correspond to the original spectra in Section 3.1. This evaluation employs the Spectral Angle Mapper (SAM) and Peak Signal-to-Noise Ratio (PSNR) metrics to capture both spectral and spatial fidelity. In the case of PSNR, the average of the different bands is computed. Furthermore, reconstruction errors are measured for each band and for each tissue class, highlighting trends present in the datasets, as discussed in Section 3.2 and Section 3.3, respectively. Second, we examine whether discriminative information is preserved after performing spectral unmixing by using the resulting abundance estimates in classification tasks and evaluating classification performance with the F1 score. The relationship between the F1 metric and the reconstruction quality is presented in Section 3.4. Qualitative assessments of the usefulness of the estimated abundances are provided in Section 3.5 in the form of colour-coded tissue classification maps.

### 3.1. Unmixing Performance

The four spectral unmixing models described were assessed on both datasets. For each patient, the reconstructed spectra were compared with the original spectra using a set of reconstruction metrics, which were then aggregated for each experimental condition (i.e., dataset × non-mixing model × normalisation). Since spectral unmixing is inherently a signal reconstruction task, the selected normalisation strategy substantially influences the outcomes. To characterise this influence, we employed three commonly used normalisation methods, $L_{2}$ normalisation, Max normalisation, and Standard Normal Variate (SNV) [4], applied to the endmembers and the raw spectra along the spectral dimension according to Equation (9).

These strategies were selected to isolate how intensity scaling affects the unmixing optimisation. $L_{2}$ normalisation preserves spectral shape while scaling to unit energy, keeping relative band ratios intact. Max normalisation divides by the peak reflectance, which standardises amplitude but can magnify noise in flat or noisy spectral regions. SNV subtracts the mean (μ) and divides by the standard deviation (σ), removing both offset and scale so the optimiser focuses purely on spectral shape. Testing all three reveals whether the unmixing models compensate for intensity variations intrinsically, or whether preprocessing must enforce them externally.

(9) $$\hat{y}=\left\{\begin{array}{cc} \frac{y}{{∥y∥}_{2}}=\frac{y}{\sqrt{\sum_{i=1}^{N}y_{i}^{2}}}\; & \left(L_{2}\right)\\ \frac{y}{\max_{i}\;\left|y_{i}\right|}\; & \left(Max\right)\\ \frac{y-\mu }{\sigma }=\frac{y-\frac{1}{N}\sum_{i=1}^{N}y_{i}}{\sqrt{\frac{1}{N}\sum_{i=1}^{N}{\left(y_{i}-\mu \right)}^{2}}}\; & \left(SNV\right) \end{array}\right.$$

**Figure 3:**
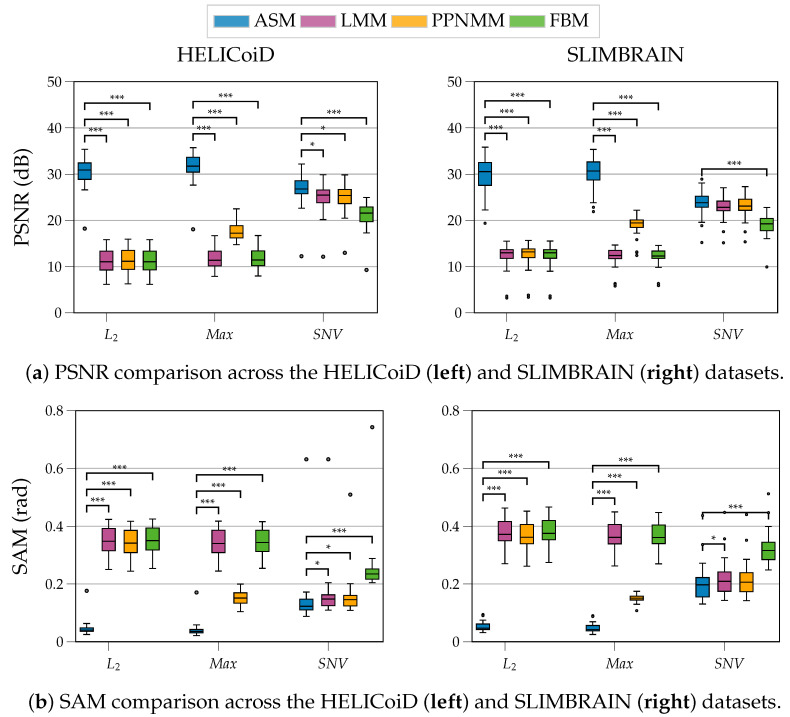
Comparison of reconstruction quality metrics for HELICoiD and SLIMBRAIN datasets. (**a**) PSNR boxplots and (**b**) SAM boxplots for both datasets. Asterisks denote statistical significance (* *p* < 0.05, *** *p* < 0.001).

The results of applying each normalisation are displayed as grouped box plots in Figure 3, where each box represents the distribution of the values of the PSNR/SAM metric for a given dataset. The lower and upper edges of the boxes correspond to the first (Q1) and third (Q3) quartiles, respectively, and the median is indicated by a horizontal black line. The whiskers extend to 1.5 × IQR (interquartile range) beyond the quartiles, while outlier data points are plotted individually as grey markers. Statistical comparisons between the proposed model (ASM) and the other spectral unmixing models were performed using the one-sided Wilcoxon signed-rank test within each normalisation method to verify the hypothesis that ASM is statistically superior. Significance levels are indicated by asterisks: * *p* < 0.05, ** *p* < 0.01, *** *p* < 0.001.

Under normalisation $L_{2}$ and Max, ASM consistently achieves PSNR values that are around 31 dB (Figure 3a), in contrast to those obtained with other methods that are around 12 dB for LMM and FBM, with the exception of PPNMM with Max, which scores near 19 dB. Similarly, this advantage is reflected almost symmetrically in median SAM values (Figure 3b), where ASM achieves values around 0.05 while competing models cluster around 0.35. An exception is again drawn for the PPNMM model under Max normalisation, a configuration that achieves a better SAM of 0.15. Nevertheless, all pairwise differences are statistically significant with p<0.001, indicating the superiority of the ASM model when these normalisations are used. The advantage of ASM under these normalisations is attributed to the scattering term: the joint optimisation of τ,α and β parameters allows the model to account for global intensity variations arising from illumination changes and specular reflections. In the absence of this term, the other models must overestimate the abundances of endmembers to capture the intensity changes present in the data, degrading the final results.

In contrast, when SNV normalisation is used, the PSNR and SAM metrics for all approaches fall within a tighter interval, ranging from ≃20–28 dB for PSNR to ≃0.1–0.3 for SAM, and the statistical distinctions between them become less evident. Interestingly, in SLIMBRAIN under SNV, ASM still maintains a statistically significant advantage over FBM (p<0.001), while the distinction from LMM and PPNMM is weaker (p<0.05). However, in HELICoiD, ASM-SNV still outperforms LMM and FBM but lacks a strong and statistically significant improvement over PPNMM. The overall reduction in variance across all models under SNV is also notable, pointing to more consistent reconstruction when SNV intensity normalisation is applied beforehand.

Overall, the ASM-Max configuration produced the most accurate reconstructions in both datasets. Upon comparison between the datasets, HELICoiD generally exhibits better reconstruction metrics than SLIMBRAIN, with a higher median PSNR, a lower median SAM, and fewer outliers, suggesting more homogeneous spectral mixing conditions. Notably, under SNV normalisation, the comparison among models is more uniform. This is consistent with the nature of SNV, which standardises each spectrum to zero mean and unit variance, thereby suppressing the intensity variations that the ASM scattering term can handle.

### 3.2. Per Wavelength Analysis

The aggregate statistics presented in Figure 3 compress spectral errors into summary measures, masking non-uniform accuracy across the wavelength domain. In practice, reconstruction accuracy is typically non-uniform across the wavelength range: some bands are recovered with high precision, while others, especially those associated with pronounced absorption features, can show domain-specific spectral deviations [36,37].

**Figure 4:**
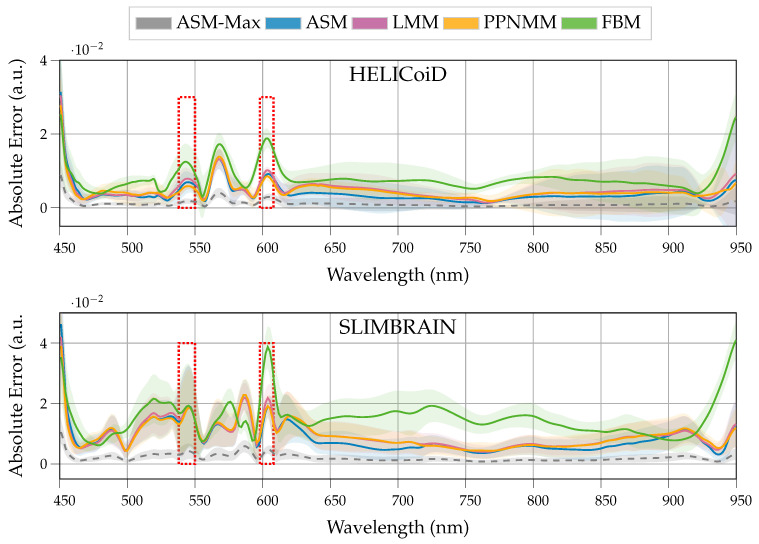
Comparison of reconstruction error per wavelength in HELICoiD (**top**) and SLIMBRAIN (**bottom**) for the different models in SNV normalisation. The best-performing overall method (ASM and max normalisation) is also included as a reference. Common peaks are marked in red dotted rectangles.

Figure 4 presents the mean absolute reconstruction error per wavelength under SNV normalisation, with shaded regions indicating the standard deviation between patients. SNV normalisation was selected for this analysis as it suppresses global intensity offsets, allowing specific band reconstruction errors to be compared more directly across models. For reference, the best performing configuration identified in Section 3.1, ASM with Max normalisation, is overlaid as a grey dashed line.

Sharp error peaks are evident at approximately 545 and 605 nm in both datasets (marked with red dashed boxes). All unmixing methods struggle at these wavelengths, though the magnitude varies across models. The consistency of these peaks across both datasets and all algorithms suggests that they reflect intrinsic tissue spectral characteristics rather than dataset-specific artefacts, arising from inter-band cross-talk, measurement-dependent spectral shifts [37], or missing endmember components. The peak at 545 nm and the elevated error around 565 nm align with the characteristic absorption bands of oxyhaemoglobin and deoxyhaemoglobin, respectively, [38], confirming haemoglobin as the dominant source of reconstruction difficulty in this spectral region. In contrast, the error feature at 605 nm corresponds to the reduced cytochrome c oxidase absorbance peak [39,40], and deviations from the expected absorbance indicate pathological alterations in mitochondrial function.

Outside these absorption bands, particularly in the 650–900 nm region, where tissue spectra are spectrally smoother, most models achieve relatively stable and low reconstruction error. A notable exception is FBM, which exhibits systematically higher error and wider confidence intervals throughout this range in both datasets. This may reflect a limitation of the bilinear interaction structure assumed by FBM in capturing the smoother, more linearly separable mixing behaviour that dominates in the near-infrared. At the spectral extremes below 470 nm and above 900 nm, the error increases across all models, consistent with a reduced signal-to-noise ratio in both camera systems at both ends of the spectrum, as characterised in Figure 2.

### 3.3. Per-Class Spectral Reconstruction Accuracy

The per-wavelength analysis identified systematic error peaks at the principal haemoglobin and reduced cytochrome c oxidase absorption bands. These findings suggest that vascular tissue, whose spectra are dominated by haemoglobin absorption, and metabolically active pathological tissue may be disproportionately responsible for reconstruction degradation. To test this hypothesis directly, we decompose the reconstruction error by tissue class, providing a sensitive diagnostic of whether the endmember library adequately represents each clinically relevant tissue category. We quantified PSNR and SAM for the three tissue categories defined by neurosurgeons: healthy tissue, tumour, and blood vessel, using ASM with Max normalisation, which demonstrated the best overall performance in both datasets. Comparable per-class trends were observed with the other model and normalisation combinations, confirming that the patterns described reflect the data structure rather than specific model behaviour.

**Figure 5:**
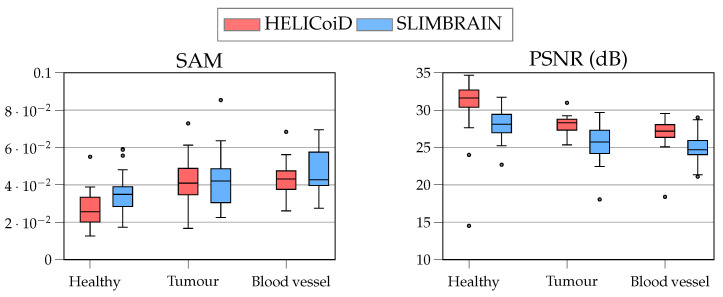
Box plot analysis of reconstruction metrics per tissue type, SAM (**left**) and PSNR (**right**) for both datasets for the ASM with max normalisation.

Figure 5 reveals consistent trends in both datasets. Healthy tissue achieves the highest reconstruction quality, with median PSNR values of approximately 32 dB for HELICoiD and 28 dB for SLIMBRAIN, and the lowest median SAM values of approximately 0.025–0.035 in both datasets. As expected from the per-wavelength analysis, the tumour and blood vessel classes show a systematic degradation of 3–5 dB in PSNR and an increase in SAM to approximately 0.04–0.045, with a notably wider interquartile range indicating greater patient-level variability within these classes.

Across all classes, SLIMBRAIN consistently yields lower median PSNR and higher median SAM than HELICoiD, with increased interquartile ranges. This systematic offset is likely to be attributable to differences in the acquisition systems, including sensor noise characteristics and different illumination choices, rather than algorithmic limitations, and is consistent with the larger performance gap observed in Figure 3.

### 3.4. Unmixing Based Segmentation

Having established that ASM with Max normalisation achieves the most accurate spectral reconstruction, we now ask whether this reconstruction fidelity translates into preserved discriminative information for tissue classification, a critical requirement for clinical utility. Rather than working with the full spectrum, a classifier here is trained exclusively on the abundance vector estimated by each unmixing model, representing a notable compression over the original spectral dimensionality.

**Figure 6:**
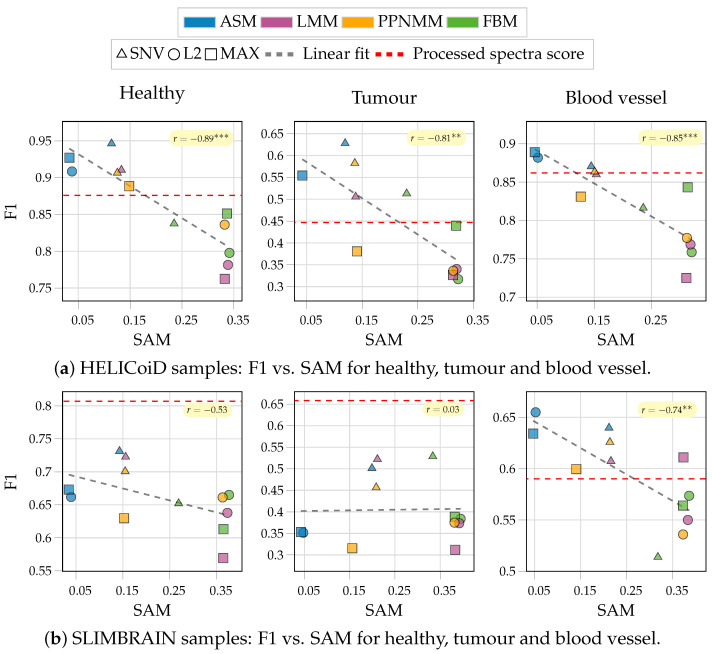
Analysis of classification performance (F1) with regard to SU reconstruction error (SAM) for HELICoiD (**a**) and SLIMBRAIN (**b**) datasets. The red lines indicate the baseline performance of training the classifier with the whole spectra after pre-processing. Asterisks denote statistical significance (** *p* < 0.01, *** *p* < 0.001).

To evaluate classification performance, we applied stratified 4-fold cross-validation at the patient level. Each fold allocated 75% of the patients to training and 25% to testing, with stratification applied to preserve the original class distribution across splits. This approach prevented data leakage from multiple captures of the same patient. For each fold, an SVM classifier was optimised using the Optuna framework over 40 trials per fold, tuning the hyperparameters *C* and γ with an RBF kernel, as demonstrated to provide the best results in a previous work [41]. Since tumour detection carries the greatest clinical importance, the optimisation objective balanced the weighted F1 score across all classes with the tumour-specific F1 score, ensuring that overall performance was not achieved at the expense of tumour misclassification. The mean F1 scores per class were calculated over the test folds for each combination of mixing model and normalisation method and are presented against the corresponding SAM reconstruction error in Figure 6. The red horizontal lines indicate the baseline F1 achieved using the full preprocessed spectra, providing a direct reference for the cost or benefit of working in the abundance domain.

The HELICoiD results (Figure 6a) demonstrate strong negative correlations between SAM and F1 in all tissue classes, confirming that a lower reconstruction error is associated with better preservation of discriminative spectral features. It is observed that the SNV normalisation consistently achieved strong classification performance, appearing towards the top part of the charts, while the other normalisations led to poorer outcomes. This indicates that the key discriminative information for tissue classification is mainly encoded in the spectral shape rather than in absolute intensity. The only exception where SNV did not outperform the other methods was for the blood vessel class, although in this case its performance was comparable to the alternative normalisations. Therefore, ASM with SNV normalisation is selected as the best overall option, achieving mean F1 scores of 0.946 (healthy), 0.629 (tumour), and 0.871 (blood vessel), compared with 0.876, 0.447 and 0.862 for the raw spectra, respectively, representing a significant improvement in tumour detection despite the substantial reduction in spectral dimensionality.

The SLIMBRAIN results (Figure 6b) present a more complex picture. The SAM-F1 relationship was weaker and more heterogeneous between tissue classes, and the tumour tissue did not show a clear monotonic trend. Mean abundance-based F1 scores, using ASM-SNV, for healthy tissue (0.767) and tumour tissue (0.525) were significantly lower than those achieved with raw spectra (0.807 and 0.658, respectively), in contrast to the improvements observed in HELICoiD. In particular, ASM with Max normalisation, the configuration that achieved the best spectral reconstruction, performed significantly worse than the raw spectra for the healthy and tumour classes (p=0.029 and p<0.001, respectively). This apparent paradox, in which the superior fidelity of the reconstruction does not translate into the superior classification performance, is an important finding. It suggests that under Max normalisation, ASM captures intensity-related variance through its scattering term, which is spectrally accurate but not discriminative for tissue classification. When SNV normalisation is applied instead, this intensity variance is suppressed at the preprocessing stage, and ASM performance no longer differs significantly from raw spectra for any class, representing the best practical compromise between spectral compression and classification fidelity. The weaker overall performance of all methods on SLIMBRAIN relative to HELICoiD is consistent with previous results and could be due to the lower SNR characteristics of that acquisition system, although further controlled analysis would be required to confirm this attribution.

Several patterns are consistent across both datasets. First, SNV normalisation emerges as the most robust preprocessing choice for classification, especially when combined with the ASM, reinforcing the conclusion that the spectral shape is more diagnostically informative than the absolute intensity in this application. Second, the tumour class consistently achieved the lowest F1 scores for all conditions and both datasets. This is attributable to two factors: the tumour category encompasses heterogeneous pathological subtypes, including multiple glioma grades and secondary tumours, which are grouped into a single class due to their limited prevalence in the data, and the high spectral variability between tumours is well documented even within the glioma subtypes [42,43], creating a classification target that is inherently difficult to represent with a fixed endmember library. Third, the class of blood vessels showed an interesting exception where the normalisation choice did not greatly affect the discriminative power of the abundances.

**Figure 7:**
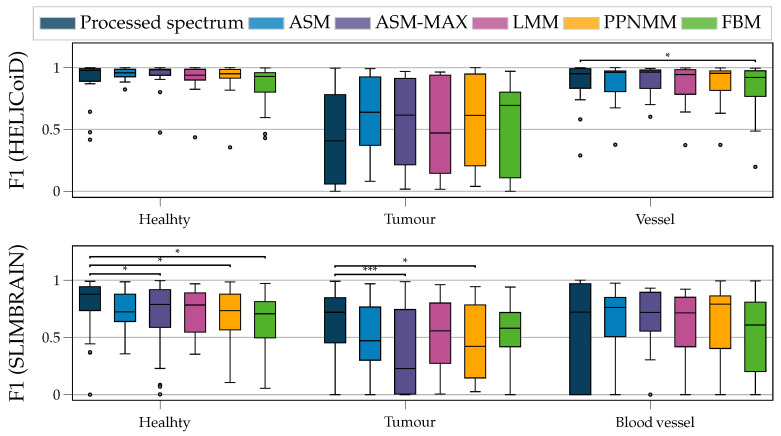
F1 scores for the SNV-based methods compared with the method that obtained the best reconstruction (ASM-Max) and the pre-processed spectra. Results from HELICoiD (**top**) and SLIMBRAIN (**bottom**) are displayed grouped by tissue type. Asterisks denote statistical significance (* *p* < 0.05, *** *p* < 0.001).

The F1 variability analysis shown in Figure 7 further contextualises these results, employing box plots to offer a more detailed view of the median values, interquartile ranges and statistical significance of the hypothesis tested. Here, the hypothesis examines whether classifiers trained on the complete processed spectral information perform better than those trained solely on the computed abundances. For HELICoiD, no statistically significant differences between classification based on the abundances and that using the pre-processed spectra were found, with the exception of FBM in blood vessels, suggesting that the abundance representation preserves classification-relevant information without significant degradation. For SLIMBRAIN, greater fold-level variability was observed across all classes, translating into a wider IQR, with significant degradation identified for ASM-Max, especially in the tumour. Nevertheless, ASM-SNV and LMM-SNV were the only configurations that showed no decrease in performance relative to the pre-processed spectrum, with no statistically significant decline observed in either dataset. Taken together with the results from Figure 6, which demonstrated the superiority of the ASM approach, this indicates that ASM-SNV is the preferred configuration when both spectral interpretability and robust classification performance are desired.

### 3.5. Qualitative Results

**Figure 8:**
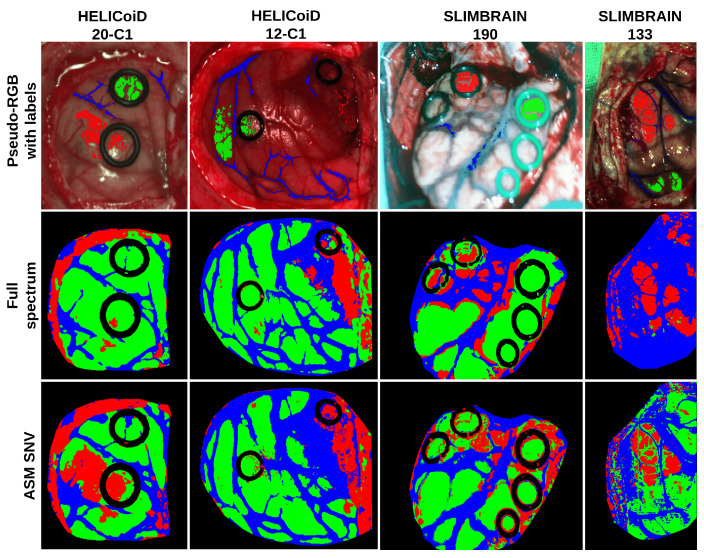
Classification results are shown for the SVM trained with pre-processed spectra (**middle row**) or the abundances (**bottom row**). The (**top row**) displays the RGB image derived from the original HSI acquisition, with the labels overlaid.

To complement the quantitative findings presented in Section 3.1, Section 3.2, Section 3.3 and Section 3.4, Figure 8 presents representative classification maps for two patients from the test of each dataset, allowing for a direct visual assessment of how abundance-based classification compares to full-spectrum classification in practice. Each patient is shown with the pseudo-RGB rendering of the HSI cube and the neurosurgeon-delineated ground truth labels (top row), the SVM classification trained on the full pre-processed spectra (middle row), and the SVM classification trained exclusively on ASM-SNV abundances (bottom row): the configuration identified as the best practical compromise between spectral compression and classification fidelity in Section 3.4.

Despite the abundance representation retaining only a small fraction of the original spectral dimensionality, the resulting classification maps are visually comparable to those obtained from the full spectrum across all four patients. However, differences at the patient-level reveal the heterogeneity that underlies the aggregate statistics. For patient 20C1 (HELICoiD) and patient 133 (SLIMBRAIN), the abundance-based classification visibly improves tumour detection relative to the raw spectrum classifier, more closely matching the neurosurgeon ground truth. This is consistent with the improved F1 tumour scores observed for ASM-SNV in HELICoiD and suggests that, for certain patients, the unmixing decomposition removes some spectral variability and exposes tumour-specific features more clearly. In contrast, for patient 12C1 (HELICoiD) and patient 190 (SLIMBRAIN), the abundance-based approach does not significantly improve tumour detection relative to raw spectra, and in some regions the classification is less precise, such as the spread of the tumour observed on the upper right image of patient 190.

Patient 133 from SLIMBRAIN is particularly interesting because the full-spectrum classifier did not detect the healthy tissue class in the image, while the abundance-based classifier successfully identified the healthy class and distinguished it from both the tumour and the blood vessel.

In conclusion, qualitative results support the idea that ASM-SNV abundance-based classification represents a viable and interpretable alternative to the full spectrum classification for intraoperative tissue classification, offering a substantial reduction in spectral dimensionality without systematic loss of diagnostic precision. The patient-level variability observed in both datasets highlights the importance of endmember library completeness and suggests that patient-adaptive or intraoperative endmember refinement could be a productive direction for improving robustness in clinical deployment.

## 4. Discussion

This study evaluated the impact of the normalisation strategy and the mixing model on spectral unmixing performance and downstream tissue classification in hyperspectral brain imaging, using two publicly available surgical datasets. The cross-dataset design allowed observed trends to be distinguished from dataset-specific artefacts, strengthening the generalisability of the conclusions drawn.

**The importance of normalisation.** The most consistent finding was that input normalisation strongly influenced both reconstruction and classification performance, as well as the choice of unmixing model itself. SNV normalisation, which standardises each spectrum to zero mean and unit variance, consistently outperformed $L_{2}$ and Max normalisation by removing global intensity offsets and forcing unmixing algorithms to focus on spectral shape. This is physically interpretable: in tissue imaging, illumination variations, specular reflections, and tissue surface geometry introduce intensity-based variance that is not related to tissue composition. By suppressing this variance, SNV allows the unmixing process to focus more on spectral features, removing a source of inter-patient variability that is otherwise transmitted to the classification, degrading the results. Vascular tissue was the exception, where normalisation $L_{2}$ performed marginally better in classification, suggesting that the intensity of haemoglobin absorption itself carries discriminative information for vessel detection that SNV partially suppresses. This normalisation-class interaction deserves further investigation, particularly given the clinical importance of identifying vascular structures during resection.

**Model complexity and the role of the scattering term.** Among the four models evaluated, ASM consistently achieved superior reconstruction fidelity under $L_{2}$ and Max normalisation, with median PSNR improvements exceeding 10–15 dB over LMM, PPNMM, and FBM. This advantage is directly attributable to the scattering correction term, which models global intensity modulation through its free parameters, allowing the abundance vector to focus on spectral shape reconstruction rather than compensating for intensity-related variance. This finding is consistent with theoretical arguments that purely linear models are insufficient for biological tissue, where multiple scattering and absorption interactions at the sub-pixel level violate the linear mixing assumption [20,44]. However, the advantage of ASM was substantially reduced under SNV normalisation, confirming that the scattering term and SNV pre-processing address a similar source of variance largely through different mechanisms. In practice, this means that when SNV is applied, the improvements of the ASM over other simpler models, such as LMM, are less notable, although ASM-SNV remained the best overall configuration for both reconstruction and classification.

**Spectral error localisation and endmember library completeness.** The per-wavelength analysis of Section 3.2 revealed that reconstruction errors are not uniformly distributed across the spectrum but concentrate at specific absorption features, most notably at approximately 545 nm and 565 nm, corresponding to the absorption peaks of oxyhaemoglobin and deoxyhaemoglobin, respectively, [38]. The persistence of these peaks across all models and both datasets confirms that they reflect a fundamental limitation of the endmember library rather than a model-specific failure. The additional error feature at approximately 605 nm was identified as an underestimated peak of the reduced cytochrome c. This cytochrome is associated with cellular respiration, and its alteration provides an important indicator of tissue health [35]. Outside these absorption bands, the 650–900 nm region showed generally stable and low reconstruction error for most models. These findings have a direct practical implication: endmember library refinement targeting the visible absorption region, particularly through inclusion of haemoglobin spectral variants across oxygenation states, is likely to yield greater improvements in reconstruction accuracy than further increases in model complexity.

**The reconstruction-classification relationship and its limits.** A key question driving this study was whether the accuracy of spectral reconstruction can predict classification performance when abundance estimates, rather than raw spectra, are used as inputs to the classifier. The findings show that the relationship is nuanced and varies across datasets. In HELICoiD, strong negative SAM-F1 correlations across all tissue classes confirmed that lower reconstruction error is associated with better preservation of discriminative information, and ASM-SNV computed abundances achieved classification performance that numerically exceeded those using the full pre-processed spectra for all classes, most notably for tumour detection. In SLIMBRAIN, this association was less pronounced and was completely missing in tumour tissue. Most strikingly, ASM with Max normalisation, the configuration that achieved the best spectral reconstruction, performed significantly worse than raw spectra for tumour and healthy tissue classification (p<0.001 and p=0.029, respectively). This seemingly paradoxical effect can be interpreted as follows: with Max normalisation, the ASM scattering term captures intensity-related variability that affects the spectral reconstruction, but this does not affect the spectral classification accuracy as it does not encode tissue-discriminative cues that the SVM classifier leverages. SNV pre-processing avoids this problem by removing intensity variance before unmixing, ensuring that neither the scattering term nor the classifier is distorted by it. This interpretation is supported by the finding that ASM-SNV did not significantly degrade classification performance relative to raw spectra in either dataset.

**Tumour classification as an inherently hard problem.** The consistently low tumour F1 scores across all conditions, models, and datasets reflect biological complexity that cannot be resolved by improved unmixing alone. The class of tumours in both HELICoiD and SLIMBRAIN encompasses multiple glioma grades and secondary tumour types, grouped due to their limited prevalence in the available data. The high intertumour spectral heterogeneity is well documented [42,43], and the fixed endmember library used here cannot capture the full range of pathological spectral variation. The weak SAM-F1 correlation for SLIMBRAIN tumours specifically suggests that for highly heterogeneous tissue, accurate reconstruction of a mean spectral signature does not guarantee preservation of the subtle spectral differences that distinguish tumour from healthy tissue at the classification level. Addressing this limitation will likely require either patient-adaptive endmember estimation or end-to-end learning frameworks that optimise endmembers directly for discrimination rather than reconstruction.

**Clinical implications and practical recommendations.** From a surgical guidance perspective, the key practical finding is that ASM-SNV abundance maps, while representing a huge spectral compression relative to the full hyperspectral cube, maintained classification performance statistically comparable to full spectrum classification in both datasets, while providing a physically interpretable decomposition of tissue composition. This reduction in dimensionality not only accelerates algorithm training but also intraoperative processing.

**Limitations.** Several limitations of this study should be acknowledged. The cohort size (26 hyperspectral captures for HELICoiD and 37 for SLIMBRAIN) limits statistical power and generalisability of the findings. We mitigated this with patient-level stratified 4-fold cross-validation to prevent data leakage, and cross-dataset testing indicates that main trends, such as SNV normalisation superiority and ASM scattering behaviour, persist across both databases. Algorithmically, the fixed endmember library cannot capture intraoperative variables like tissue dehydration, blood pooling, or surgical agents, nor does it model gradual spectral transitions in tumour infiltration zones. Addressing these limitations will likely require moving beyond rigid per-pixel decomposition to spatially constrained unmixing, endmember variability modelling [45,46], and task-driven unmixing frameworks that jointly optimise spectral reconstruction and downstream classification, allowing the estimated abundance maps to be directly optimised for tissue discrimination rather than reconstruction fidelity alone [17]. Ground truth also relies on neurosurgeon annotations rather than histopathology, introducing unquantified inter-annotator variability and annotation uncertainty. For clinical translation, hyperspectral unmixing currently lacks the prospective multi-centre trials, standardised acquisition protocols, and histopathologically validated labels that would be expected before clinical translation and regulatory evaluation. Bridging these algorithmic, clinical, and hardware gaps remains necessary before intraoperative deployment as a certified medical device.

## Figures and Tables

**Table 1:** Summary of the patient cohort per database.

Characteristic	HELICoiD	SLIMBRAIN
Captures (n)	26	37
Captures with tumour	15 (58%)	22 (59%)
Total labelled pixels	197,550	1,135,771
Class pixel distribution		
Healthy	36.2%	53.1%
Tumour	8.7%	7.2%
Blood vessel	55.1%	39.7%

## Data Availability

The data presented in this study are available upon request for both databases. Refer to [23] for HELICoiD and [24] for SLIMBRAIN data.

## Appendix A. Patient Selection

The cohort from the HELICoiD database comprised 26 patients: 12C1, 13C1, 20C1, 21C1, 22C1, 27C2, 35C1, 36C1, 37C1, 38C1, 39C1, 40C1, 41C1, 41C2, 42C1, 42C2, 43C1, 43C2, 4C2, 50C1, 54C1, 55C1, 56C1, 57C1, 7C1 and 8C1. Of these, 15 patients (58%) presented tumour tissue in their labelled regions, while 11 cases contained only healthy and vascular tissue.

The cohort from the SLIMBRAIN database included 37 patients: 120C01, 125C01, 126C01, 130C01, 133C01, 133C02, 135C01, 136C01, 138C01, 141C01, 145C01, 147C01, 148C01, 149C01, 150C01, 154C01, 158C01, 162C01, 167C01, 168C01, 174C01, 177C01, 181C01, 183C01, 187C01, 188C01, 189C01, 190C01, 192C01, 194C01, 195C01, 199C01, 201C01, 202C01, 203C01, 204C01 and 205C01. Tumour tissue was present in 22 patients (59%), with the remaining 15 cases showing only non-pathological tissue types.
